# Discovering novel plasma biomarkers for ischemic stroke: Lipidomic and metabolomic analyses in an aged mouse model

**DOI:** 10.1016/j.jlr.2024.100614

**Published:** 2024-08-02

**Authors:** Danielle A. Becktel, Jennifer B. Frye, Elizabeth H. Le, Susan A. Whitman, Rick G. Schnellmann, Helena W. Morrison, Kristian P. Doyle

**Affiliations:** 1 Department of Immunobiology, College of Medicine, University of Arizona, Tucson, Arizona, USA; 2 Department of Pharmacology and Toxicology, R. Ken Coit College of Pharmacy, University of Arizona, Tucson, Arizona, USA; 3 Coit Center for Longevity and Neurotherapeutics, R. Ken Coit College of Pharmacy, University of Arizona, Tucson, Arizona, USA; 4 BIO5 Institute, College of Medicine, University of Arizona, Tucson, Arizona, USA; 5 College of Nursing, University of Arizona, Tucson, Arizona, USA; 6 Department of Neurology, College of Medicine, University of Arizona, Tucson, Arizona, USA; 7 Arizona Center on Aging, University of Arizona, Tucson, Arizona, USA; 8 Department of Psychology, College of Science, University of Arizona, Tucson, Arizona, USA; 9 Department of Neurosurgery, College of Medicine, University of Arizona, Tucson, Arizona, USA

**Keywords:** arachidonic acid, brain lipids, inflammation, lipids, lipoxygenase, sphingolipids, 12-HETE, neurodegeneration, neurofilament light, myelin

## Abstract

Ischemic stroke remains a leading cause of mortality and long-term disability worldwide, necessitating efforts to identify biomarkers for diagnosis, prognosis, and treatment monitoring. The present study aimed to identify novel plasma biomarkers of neurodegeneration and inflammation in a mouse model of stroke induced by distal middle cerebral artery occlusion. Using targeted lipidomic and global untargeted metabolomic profiling of plasma collected from aged male mice 24 h after stroke and weekly thereafter for 7 weeks, we discovered distinct acute and chronic signatures. In the acute phase, we observed elevations in myelin-associated lipids, including sphingomyelin (SM) and hexosylceramide (HCER) lipid species, indicating brain lipid catabolism. In the chronic phase, we identified 12-hydroxyeicosatetraenoic acid (12-HETE) as a putative biomarker of prolonged inflammation, consistent with our previous observation of a biphasic pro-inflammatory response to ischemia in the mouse brain. These results provide insight into the metabolic alterations detectable in the plasma after stroke and highlight the potential of myelin degradation products and arachidonic acid derivatives as biomarkers of neurodegeneration and inflammation, respectively. These discoveries lay the groundwork for further validation in human studies and may improve stroke management strategies.

Ischemic stroke, a leading cause of mortality and long-term disability worldwide, affects approximately 13.7 million people annually and was responsible for 5.5 million deaths in 2019 (1, 2, 3). This acute cerebrovascular event results from the sudden reduction or cessation of blood flow to a region of the brain, causing immediate loss of neurological function and triggering an ischemic cascade that leads to permanent brain damage (4).

Clinical treatment of ischemic stroke is currently focused on restoring cerebral blood flow through pharmacological thrombolytic therapy with recombinant tissue plasminogen activator (rt-PA) or endovascular therapy with mechanical thrombectomy (5, 6). However, these interventions are limited by narrow therapeutic time windows and contraindications. Given these limitations, there is a critical need for reliable and widely applicable biomarkers for timely diagnosis and prognosis.

To address this need, researchers have investigated several biomarkers, including S100 calcium-binding protein B (S100B), glial fibrillary acidic protein (GFAP), C-reactive protein (CRP), D-dimer, and more recently, neurofilament light (NfL) (7, 8). While these biomarkers have been linked to various aspects of stroke pathology and outcomes, their utility is constrained by a lack of specificity. Moreover, these biomarkers have been predominantly studied in the acute phase after stroke. Given these constraints, there is a need for more specific biomarkers to precisely monitor stroke recovery and predict long-term outcomes. Plasma biomarkers hold considerable promise due to the minimally invasive nature of sample collection and the potential to reflect dynamic physiological and pathological processes occurring in the brain after stroke (7). This approach could provide valuable insights into both the acute and chronic phases of stroke recovery.

In recent years, the role of lipid metabolism in stroke pathophysiology has garnered increasing attention, with alterations in lipid profiles linked to stroke risk, severity, and outcomes (9, 10). Building on this understanding, our previous research, using a mouse model of stroke induced by distal middle cerebral artery (MCA) occlusion, revealed a biphasic pro-inflammatory cytokine response to ischemia in the brain, seemingly driven by dysregulated lipid metabolism in the stroke infarct. This inflammatory response was characterized by an initial phase occurring within 24 h to 1 week after stroke, followed by a more sustained phase between 4 weeks to 8 weeks, which coincided with the accumulation of foamy macrophages and lipid droplets (11). Importantly, the lipids within these foamy macrophages likely originate from the breakdown of myelin and other brain lipids, indicating overwhelmed lipid processing by phagocytes in the chronic phase after stroke.

Using targeted lipidomic profiling of mouse stroke infarcts, we have also previously identified alterations in specific lipid classes, signifying myelin breakdown and processing within immune cells (12). Nevertheless, comprehensive temporal profiling of lipid and metabolite alterations in plasma, particularly in the chronic phase after stroke, remains limited.

Considering this knowledge gap, we hypothesized that the plasma lipidome and metabolome would exhibit an acute signature of brain lipid catabolism and a chronic signature of dysregulated lipid metabolism after stroke. To test this hypothesis, we conducted targeted lipidomic and global untargeted metabolomic profiling of plasma collected from aged male mice 24 h, 1 week, 2 weeks, 3 weeks, 4 weeks, 5 weeks, 6 weeks, and 7 weeks after stroke, using naive mice as controls. Concurrently, we quantified plasma NfL levels to determine whether these lipids and metabolites align with neuroaxonal damage, offering an integrated view of molecular alterations in the plasma after stroke.

## Materials and Methods

### Animals

Aged (20- to 23-month-old) wild-type male C57BL/6 mice were obtained from the National Institute on Aging. Mice were housed in a temperature-controlled suite with a 12-h light–dark regimen and *ad libitum* access to food and water. All experimental procedures were conducted in accordance with the animal care standards of the National Institutes of Health and approved by the University of Arizona Institutional Animal Care and Use Committee. Upon termination of each experiment, mice were anesthetized by isoflurane inhalation and secured in a supine position. For terminal intracardiac bleeding, the thoracic cavity was sterilized with 70% ethanol, and a midline incision was performed to expose the heart. A sterile syringe, equipped with a fine-gauge needle, was inserted into the left ventricle of the heart, and blood was drawn by gentle aspiration. Following exsanguination by intracardiac bleeding, mice were perfused with 0.9% saline. Collected blood samples were immediately placed on ice in EDTA-coated microcentrifuge tubes and centrifuged at 4°C for 10 min at 5,000 *g* to separate plasma. Plasma samples were stored at −80°C until further analysis.

### Retro-orbital bleeding

A heparinized microhematocrit capillary tube was gently inserted into the retro-orbital sinus, positioned at the medial canthus of the eye. Blood was collected by capillary action into an EDTA-coated microcentrifuge tube. Collected blood samples were immediately placed on ice and centrifuged at 4°C for 10 min at 5,000 *g* to separate plasma. Plasma samples were stored at −80°C until further analysis.

### Stroke surgeries

Stroke was induced in mice using the distal MCA occlusion + hypoxia (DH) model. The DH stroke model generates a sizable infarct (24% of the ipsilateral hemisphere centered on the somatosensory cortex), has low variability, and has exceptional long-term survivability. The addition of hypoxia is necessary because C57BL/6 mice that undergo DH stroke without hypoxia have much smaller infarcts. The methodology of the DH stroke model and validation of the controls have been previously published (13, 14). To induce stroke, mice were anesthetized by isoflurane inhalation and kept at 37°C throughout the surgical procedure. For all experiments, mice were injected subcutaneously (s.c.) with a single dose of buprenorphine hydrochloride (0.1 mg/kg) dissolved in sterile saline. Following pre-operative preparation, an incision was made to expose the right temporoparietal skull between the orbit and the ear. Under an operating microscope, a small hole was made with a high-speed microdrill through the outer surface of the semi-translucent skull over the visually identified MCA at the level of the parietal cerebral artery. Permanent occlusion of the MCA was performed by electrocoagulation with a small vessel cauterizer. Surgical wounds were closed using Surgi-lock 2oc tissue adhesive. Mice were then immediately transferred to a hypoxia chamber containing 11% oxygen and 89% nitrogen for 45 min. Extended-release buprenorphine (Ethiqa XR, 3.25 mg/kg s.c.) was administered 24 h after surgery as post-operative analgesia.

### MRI

Infarct, ventricle, and hippocampus volumes were assessed in a subset of mice by MRI using a Bruker BioSpec 70/20 7.0T scanner with ParaVision-360.3.2 software and a 4-channel phase array mouse coil. Mice were placed in a cradle equipped with a stereotaxic frame, an integrated heating system to maintain body temperature at 37 ± 1°C, and a pressure probe to monitor respiration. During MRI acquisition, anesthesia was maintained by inhalation of 1.5%–3% isoflurane. High-resolution structural images were acquired using a *T*_2_-weighted RARE Bruker pulse sequence with the following parameters: repetition time (*TR*) = 2,500 ms; flip angle = 30°; RARE factor = 8; matrix size = 256 × 256; averages = 2; field of view = 20 mm × 20 mm; slice thickness = 0.8 mm; number of slices = 15; acquisition time = 2 min 40 s. Infarcts, ventricles, hippocampi, and hemispheric cross-sections were manually delineated on *T*_2_-weighted MR images using Mango v4.1.

### Plasma neurofilament light quantification (Quanterix®)

NfL was quantified using the Simoa® (Single Molecule Array) NF-Light v2 Advantage Assay (Quanterix®, Cat. No. 104073) according to manufacturer instructions. Each control, calibrator, and experimental plasma sample was measured in duplicate on the Simoa® SR-X Instrument. In the two-step immunoassay, paramagnetic beads, pre-coated with an anti-NfL antibody, are incubated with the sample and biotinylated detector antibody. Target molecules present in the sample are captured by the antibody-coated beads and bind with the biotinylated detector antibody simultaneously. Following a wash, a conjugate of streptavidin-ß-galactosidase (SBG) is mixed with the beads. SBG binds to biotinylated detector antibodies, labeling captured targets. Following a final wash, the beads are resuspended in a resorufin ß-D-galactopyranoside (RGP) substrate solution and transferred to the Simoa® disc. Individual beads are then sealed within microwells in the array. If the target has been captured and labeled on the bead, ß-galactosidase hydrolyzes the RGP substrate into a fluorescent product that provides the signal for measurement. A single labeled target molecule results in sufficient fluorescent signal in 30 s to be detected and counted by the Simoa® optical system.

### Plasma targeted lipidomic and global untargeted metabolomic analyses (Metabolon, Inc.)

Plasma samples for targeted lipidomic and global untargeted metabolomic analyses were transported on dry ice to Metabolon, Inc. and subsequently preserved at −80°C. Targeted lipidomics specifically detects lipids from a predetermined panel. Untargeted metabolomics is a comprehensive technique that impartially and systematically detects and identifies metabolites within a biological sample. Metabolomics generally captures polar, water-soluble metabolites, including polar lipids (15).

Targeted lipidomic analysis was performed using differential mobility spectroscopy. Differential mobility spectroscopy not only distinguishes species based on variations in mass-to-charge ratio (*m/z*) and retention time/index (RI) but also considers size and shape, thereby enabling the identification of closely related lipid species. Briefly, lipids were extracted from plasma in the presence of deuterated internal standards by butanol-methanol extraction (16), concentrated under nitrogen, and reconstituted in dichloromethane:methanol solution containing ammonium acetate. Samples were analyzed via both positive and negative mode electrospray MS. Individual lipid species were quantified by multiplying peak area ratios of target compounds and their assigned internal standards by the internal standard concentration in the sample. Each lipid class concentration was calculated by summing all lipids belonging to that class. Each fatty acid composition was calculated through the proportion of each class composed of individual fatty acids.

Global untargeted metabolomic analysis was performed using ultrahigh-performance liquid chromatography coupled with tandem mass spectrometry (UPLC-MS/MS). Briefly, recovery and internal standards were added to plasma samples for evaluation of extraction efficiency and instrument performance, respectively. Metabolites were extracted with methanol and analyzed by reverse-phase (RP)/UPLC-MS/MS (positive and negative ion modes) and hydrophilic interaction liquid chromatography (HILIC)/UPLC-MS/MS. Metabolites were identified by automated ion peak comparison from each sample to library entries of purified standards or recurrent unknown entities. Metabolon, Inc. maintains a reference library based on authenticated standards that contain the RI, *m/z*, and fragmentation data for all molecules. Biochemical identifications were based on three criteria: RI within a narrow window of the proposed identification, accurate mass switch to the library ± 10 ppm, and the MS/MS forward and reverse scores between the experimental data and authentic standards. Each metabolite within a biological sample was quantified by area under the curve and normalized to account for day-to-day variation.

### Statistical analyses

Statistical analyses were performed using GraphPad Prism 10.0. Data were tested first for outliers with the ROUT method (Q = 1%) and then for normal distribution with the Shapiro-Wilk (W) test. For data demonstrating normal distribution, parametric tests were applied, specifically: (1) a two-tailed, unpaired *t-*test for comparisons between two groups, or (2) an ordinary one-way ANOVA followed by Dunnett’s multiple comparisons test for comparisons between multiple groups. In cases where data did not exhibit normal distribution, nonparametric tests were utilized: (1) a Mann-Whitney *U* test for comparisons between two groups, or (2) a Kruskal–Wallis test followed by Dunn’s multiple comparisons test for comparisons between multiple groups. Spearman correlation analysis was conducted to assess the association between MRI parameters, and results were represented graphically using curves indicating 95% confidence intervals. Mean with SD was reported for NfL analysis. In all other analyses, data were presented as box plots, where the boxes depict the 25^th^ to 75^th^ percentile range, whiskers denote the minimum to maximum data span, and horizontal lines indicate the median value. Individual values for each mouse are plotted on their respective graphs.

MetaboAnalyst 5.0 was used to create volcano plots and heatmaps from lipid species concentrations and metabolite peak areas. Prior to analysis, a variance filter (i.e., SD) was applied, filtering out 20% of all features. Subsequently, data underwent normalization by sum, followed by log transformation. Auto-scaling was employed for data scaling. Hierarchical clustering heatmaps were generated, offering an intuitive visualization of log-normalized concentrations. Euclidean correlations and Ward clustering algorithms were used to measure distances between data points. Volcano plots were generated using a fold change (FC) threshold of 2.0 and false discovery rate (FDR)-adjusted *P* value threshold of 0.05. These comprehensive analyses provide insights into the distribution, patterns, and relationships within the lipid and metabolite datasets.

## Results

### NfL is a plasma biomarker of neurodegeneration after stroke in aged mice

**Fig. 1:**
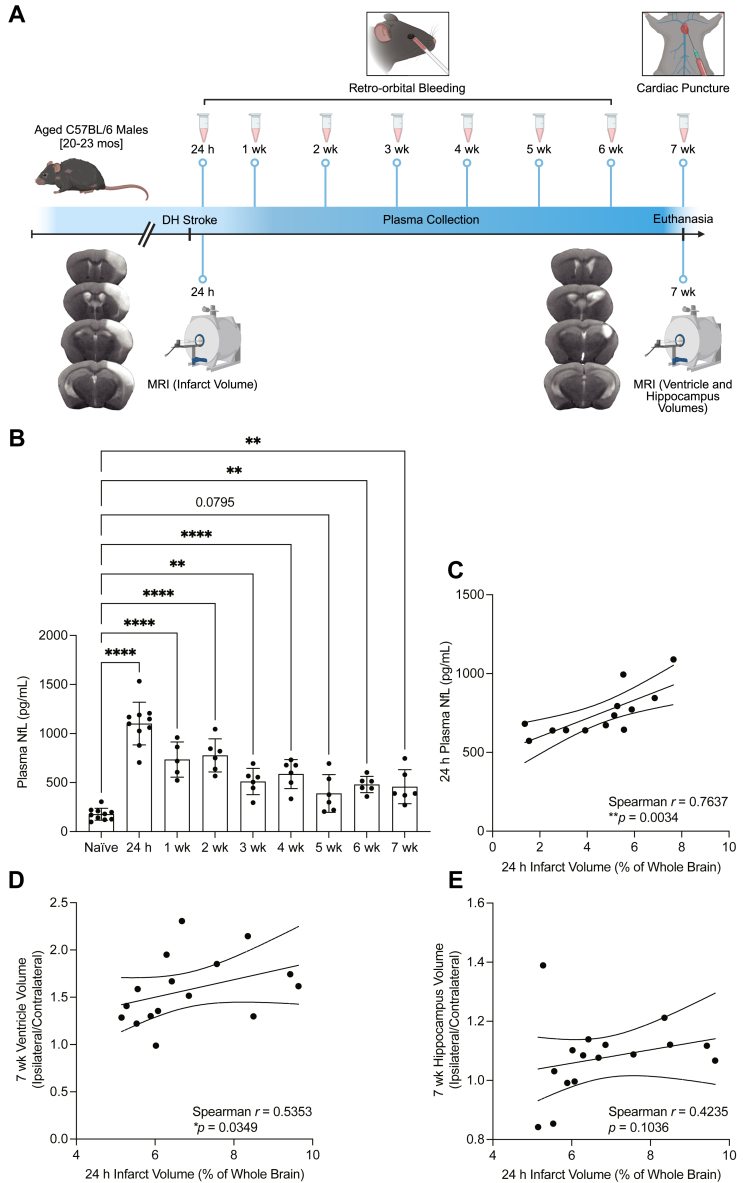
NfL is a plasma biomarker of neurodegeneration after stroke in aged mice. A: Experimental design, 20- to 23-month-old male mice were subjected to distal middle cerebral artery occlusion + hypoxia (DH) stroke. Plasma was collected 24 h after stroke and weekly thereafter for 7 weeks. *T*_2_-weighted MRI was performed on a subset of mice at 24 h and 7 weeks after stroke to assess acute and chronic pathological sequelae (i.e., infarct expansion, hippocampal edema, and ventricle enlargement). Representative *T*_2_-weighted MR images captured 24 h after stroke illustrate an infarct centered on the somatosensory cortex, extending to the corpus callosum. Representative *T*_2_-weighted MR images captured 7 weeks after stroke illustrate enlargement of the ipsilateral ventricle and hippocampus. B: Plasma NfL levels remain elevated for at least 7 weeks after stroke in aged mice (n = 5–10; ANOVA, ∗∗∗∗*P* < 0.0001; Dunnett’s multiple comparisons test, ∗∗*P* < 0.01, ∗∗∗∗*P* < 0.0001). Data are presented as mean ± SD. C: Plasma NfL levels significantly correlated with infarct volume 24 h after stroke (n = 13; Spearman *r* = 0.7637, ∗∗*P* < 0.01). D: Infarct volume 24 h after stroke significantly correlated with ventricle enlargement 7 weeks after stroke (n = 16; Spearman *r* = 0.5353, ∗*P* < 0.05). E: Infarct volume 24 h after stroke weakly correlated with hippocampal edema 7 weeks after stroke (n = 16; Spearman *r* = 0.4235, *P* > 0.05). Curved lines represent 95% confidence bands for the linear fit.

NfL has emerged as a prospective biomarker for ischemic stroke, reflecting the dynamic and ongoing process of neuroaxonal damage (17). To assess the temporal dynamics of NfL accumulation in the plasma after stroke, blood sample collections were performed at 24 h, 1 week, 2 weeks, 3 weeks, 4 weeks, 5 weeks, 6 weeks, and 7 weeks after induction of DH stroke in aged (20- to 23-month-old) male mice. Concomitantly, *T*_2_-weighted MRI was conducted at 24 h to assess infarct expansion and at 7 weeks to assess hippocampal edema and lateral ventricle enlargement as measures of chronic neurodegeneration (Fig. 1A). We discovered that NfL was significantly elevated in the plasma of aged male mice at 24 h, 1 week, 2 weeks, 3 weeks, 4 weeks, 6 weeks, and 7 weeks after DH stroke compared to naïve controls (Fig. 1B). These results are in accordance with clinical studies demonstrating elevated levels of serum NfL in the first few days after stroke onset and in follow-up assessments at 3 months (18). We also observed a significant positive correlation between plasma NfL levels and infarct volumes 24 h after stroke (Fig. 1C).

To further evaluate the connection between acute and chronic pathological sequelae, Spearman correlation analyses were performed on MRI-based volumetric measures. We discovered a significant positive correlation between infarct volumes 24 h after stroke and lateral ventricle volumes 7 weeks after stroke (Fig. 1D). In addition, we observed a positive correlation between infarct volumes 24 h after stroke and hippocampus volumes 7 weeks after stroke; however, the relationship was not significant (Fig. 1E).

### Plasma lipidome 24 h after stroke in aged mice

**Fig. 2:**
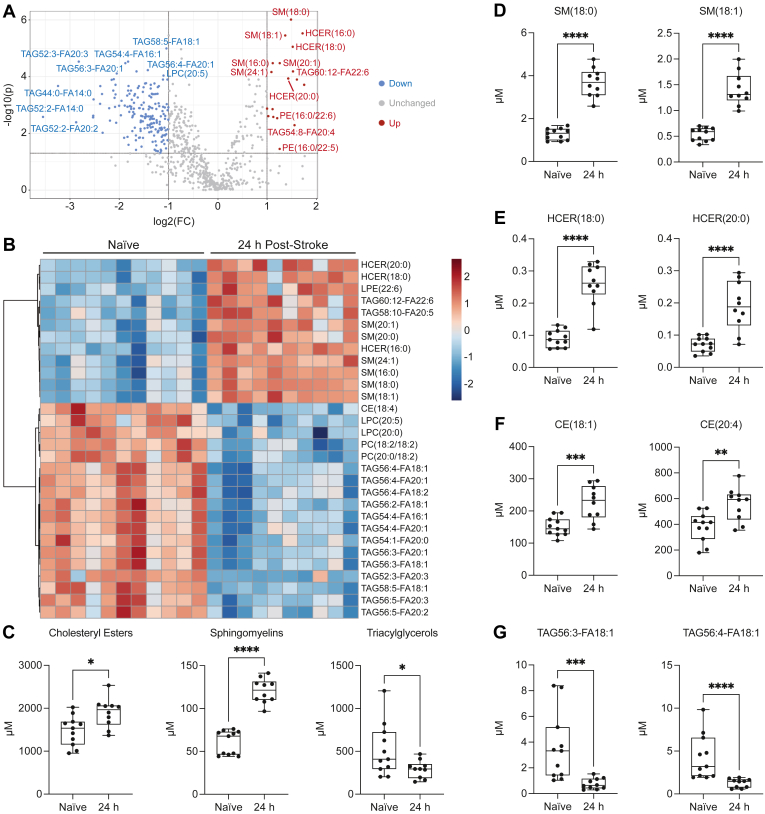
Plasma lipidome 24 h after stroke in aged mice. A: Volcano plot illustrating significant differences in lipid abundance between plasma from naïve mice and mice 24 h after stroke (FDR-adjusted *P* < 0.05; FC > |2|). B: Hierarchical clustering heatmap depicting the top 30 plasma lipids ranked by *t*-test (Euclidean; Ward). C: Lipid classes, reported as summations of all species of CE, SM, and TAG, respectively, are significantly altered in the plasma 24 h after stroke (CE: n = 10–11; unpaired *t*-test, ∗*P* < 0.05; SM: n = 10–11; Mann-Whitney *U* test, ∗∗∗∗*P* < 0.0001; TAG: n = 10–11; unpaired *t*-test, ∗*P* < 0.05). D: SM are significantly elevated in the plasma 24 h after stroke (n = 10–11; unpaired *t*-tests, ∗∗∗∗*P* < 0.0001). E: HCER are significantly elevated in the plasma 24 h after stroke (n = 10–11; unpaired *t*-tests, ∗∗∗∗*P* < 0.0001). F: CE are significantly elevated in the plasma 24 h after stroke (n = 10–11; unpaired *t*-tests, ∗∗*P* < 0.01, ∗∗∗*P* < 0.001). G: TAG are significantly reduced in the plasma 24 h after stroke (n = 10–11; Mann-Whitney *U* tests, ∗∗∗*P* < 0.001, ∗∗∗∗*P* < 0.0001). Data are presented as box and whisker plots, with boxes extending from the 25^th^ to 75^th^ percentiles and whiskers extending from the minimum to maximum values.

To determine whether the plasma lipidome expresses a signature of brain lipid catabolism in the acute phase after stroke, plasma samples were collected from aged (20- to 23-month-old) male mice 24 h after stroke and from naïve mice. These samples were sent to Metabolon, Inc. for targeted lipidomic analysis using the Complex Lipid Panel (CLP), which provides quantitation of up to 1,100 individual lipid species from 14 lipid classes. The resultant volcano plot illustrates the distribution of 18 increased and 177 decreased lipid species. These altered lipid species consisted primarily of hexosylceramides (HCER), sphingomyelins (SM), phosphatidylethanolamines (PE), and triacylglycerols (TAG), broadly indicating an increase in sphingolipids and phospholipids and a decrease in neutral lipids 24 h after stroke (Fig. 2A). To further assess these alterations in an unbiased manner, a hierarchical clustering heatmap was constructed to depict the top 30 lipids ranked by *t-*test. Consistent with the volcano plot, the heatmap illustrates an elevation in HCER and SM lipid species and a reduction in lysophosphatidylcholine (LPC), phosphatidylcholine (PC), and TAG lipid species 24 h after stroke (Fig. 2B). Upon assessment of lipid classes, which were quantified by summing categories of lipids, we discovered that cholesteryl esters (CE) and SM were increased, and TAG was decreased in the plasma 24 h after stroke (Fig. 2C). These alterations were confirmed in analyses of individual lipid species, including SM(18:0), SM(18:1), HCER(18:0), HCER(20:0), CE(18:1), CE(20:4), TAG56:3-FA18:1, and TAG56:4-FA18:1 (Fig. 2D–G). These results indicate that there is a distinct signature of lipid catabolism in the plasma lipidome 24 h after stroke, with a substantial proportion of altered lipids constituting essential components of myelin. Notably, nearly all quantified SM and HCER lipid species were significantly elevated in the plasma 24 h after stroke (supplemental Table S1).

### Temporal profile of the plasma lipidome after stroke in aged mice

**Fig. 3:**
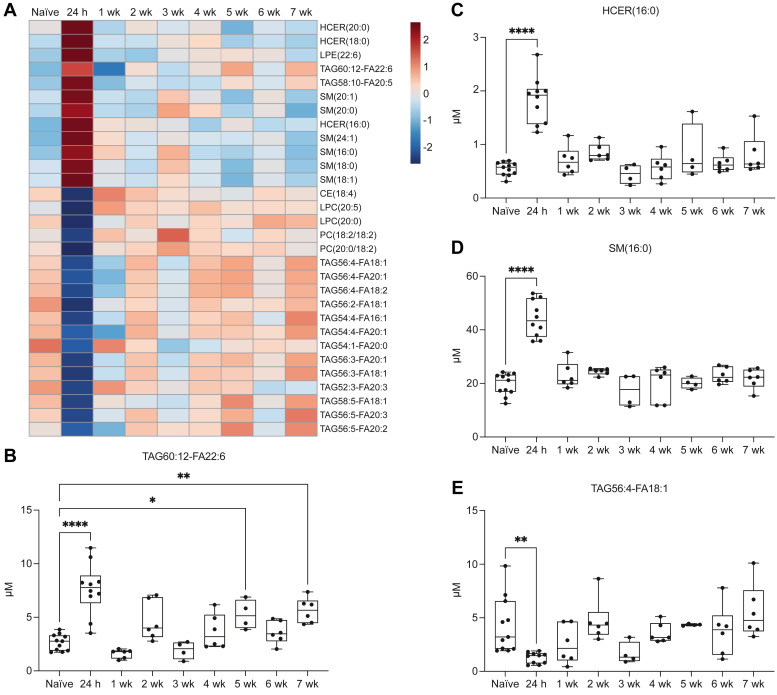
Temporal profile of the plasma lipidome after stroke in aged mice. A: Heatmap depicting group averages of the top 30 plasma lipids altered 24 h after stroke compared to naïve controls. B: TAG60:12-FA22:6 is significantly elevated in the plasma 24 h, 5 weeks, and 7 weeks after stroke (n = 4–11; ANOVA, ∗∗∗∗*P* < 0.0001; Dunnett’s multiple comparisons test, ∗*P* < 0.05, ∗∗*P* < 0.01, ∗∗∗∗*P* < 0.0001). C: HCER(16:0) is transiently elevated in the plasma 24 h after stroke (n = 4–11; Kruskal-Wallis test, ∗∗∗∗*P* < 0.0001; Dunn’s multiple comparisons test, ∗∗∗∗*P* < 0.0001). D: SM (16:0) is transiently elevated in the plasma 24 h after stroke (n = 4–11; ANOVA, ∗∗∗∗*P* < 0.0001; Dunnett’s multiple comparisons test, ∗∗∗∗*P* < 0.0001). E: TAG56:4-FA18:1 is transiently reduced in the plasma 24 h after stroke (n = 4–11; Kruskal-Wallis test, ∗∗∗*P* < 0.001; Dunn’s multiple comparisons test, ∗∗*P* < 0.01). Data are presented as box and whisker plots, with boxes extending from the 25^th^ to 75^th^ percentiles and whiskers extending from the minimum to maximum values.

To assess whether the acute signature of brain lipid catabolism was sustained in the weeks after stroke, plasma samples were collected at 1 week, 2 weeks, 3 weeks, 4 weeks, 5 weeks, 6 weeks, and 7 weeks after stroke in aged (20- to 23-month-old) male mice. These samples were also sent to Metabolon, Inc., for targeted lipidomic analysis using the CLP. To visualize the temporal profiles of the top 30 lipids altered 24 h after stroke, the hierarchical clustering heatmap in Fig. 2B was expanded to include the subacute and chronic phases after stroke. The resultant heatmap demonstrates that the acute alterations in the plasma lipidome 24 h after stroke are transient; within 1 week after stroke, most plasma lipids have reverted to baseline levels (Fig. 3A). Interestingly, the temporal profile of TAG60:12-FA22:6 was biphasic, with acute elevation at 24 h and chronic elevation at 5 weeks and 7 weeks after stroke (Fig. 3B). However, the temporal profiles of HCER(16:0), SM(16:0), and TAG56:4-FA18:1 were monophasic, with acute alterations 24 h after stroke that returned to baseline levels by 1 week (Fig. 3C–E). These results indicate that there is a distinct signature of brain lipid catabolism in the plasma in the acute phase after stroke; however, these circulating lipids return to baseline levels within 1 week.

### Plasma metabolome 24 h after stroke in aged mice

**Fig. 4:**
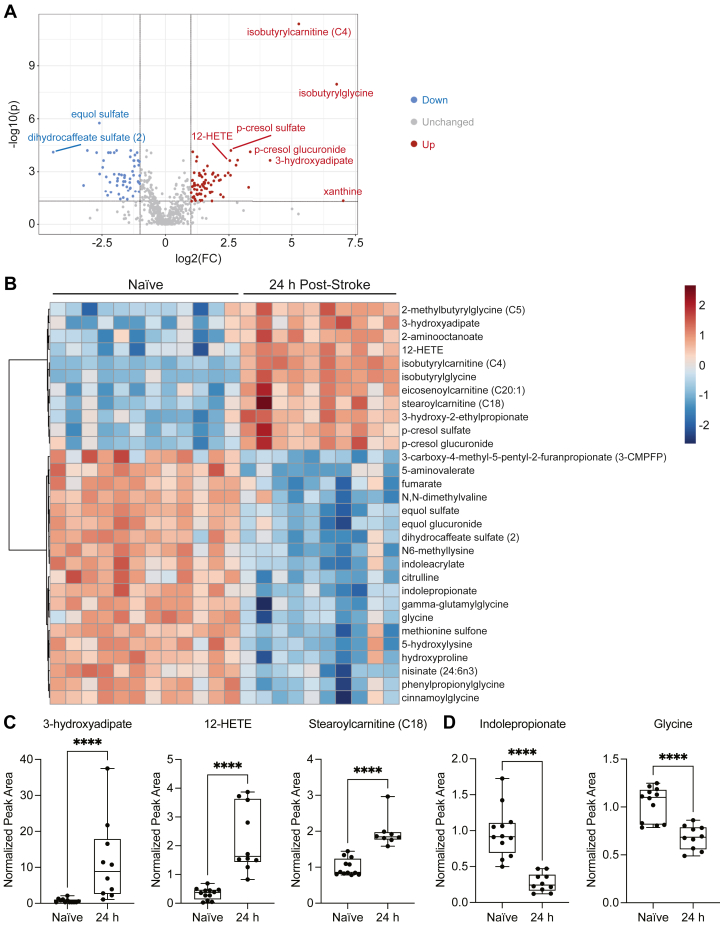
Plasma metabolome 24 h after stroke in aged mice. A: Volcano plot illustrating significant differences in metabolite abundance between plasma from naïve mice and mice 24 h after stroke (FDR-adjusted *P* < 0.05; FC > |2|). B: Hierarchical clustering heatmap depicting the top 30 plasma metabolites ranked by *t*-test (Euclidean; Ward). C and D: Lipid metabolism, represented by 3-hydroxyadipate, 12-HETE, and stearoylcarnitine (C18), and amino acid metabolism, represented by indolepropionate and glycine, are altered 24 h after stroke (12-HETE, indolepropionate, glycine: n = 10–12; unpaired *t*-tests, ∗∗∗∗*P* < 0.0001; 3-hydroxyadipate, stearoylcarnitine (C18): n = 8–12; Mann-Whitney *U* test, ∗∗∗∗*P* < 0.0001). Data are presented as box and whisker plots, with boxes extending from the 25^th^ to 75^th^ percentiles and whiskers extending from the minimum to maximum values.

To determine whether the plasma metabolome reflects a concomitant signature of dysregulated lipid metabolism in the acute phase after stroke, additional plasma samples were collected from aged (20- to 23-month-old) male mice 24 h after stroke and from naïve mice. These samples were sent to Metabolon, Inc. for global untargeted metabolomic analysis using the Global Discovery Panel, which provides an assessment of more than 5,400 metabolites across 70 metabolic pathways, including amino acid metabolism, energy metabolism, and lipid metabolism. The resultant volcano plot illustrates the distribution of 73 increased and 59 decreased metabolites (Fig. 4A). To further assess these alterations in an unbiased manner, a hierarchical clustering heatmap was constructed to depict the top 30 metabolites ranked by *t-*test. Consistent with the volcano plot, the heatmap illustrates an elevation in metabolites involved in lipid metabolism, including 3-hydroxyadipate, 12-HETE, and stearoylcarnitine (C18), and a reduction in metabolites involved in amino acid metabolism, including N6-methyllysine, indolepropionate, and glycine, 24 h after stroke (Fig. 4B). These alterations were confirmed in analyses of individual metabolites, including 3-hydroxyadipate, 12-HETE, stearoylcarnitine (C18), indolepropionate, and glycine (Fig. 4C-D).

Based on the signature of brain lipid catabolism in the plasma lipidome 24 h after stroke, we hypothesized that metabolites involved in lipid metabolism, such as fatty acids, acyl carnitines, and eicosanoids, would be elevated in the plasma 24 h after stroke. Accordingly, the elevation of 3-hydroxyadipate, a dicarboxylic acid, and 2-aminooctanoate, an α-amino fatty acid, signifies fatty acid mobilization in the plasma. In addition, we observed an increase in fatty acid metabolism, indicated by the elevation of eicosenoylcarnitine (C20:1), a monounsaturated acyl carnitine, and stearoylcarnitine (C18), a long chain saturated acyl carnitine. Notably, nearly all quantified acyl carnitines were elevated in the plasma 24 h after stroke (supplemental Table S2).

### Temporal profile of the plasma metabolome after stroke in aged mice

**Fig. 5:**
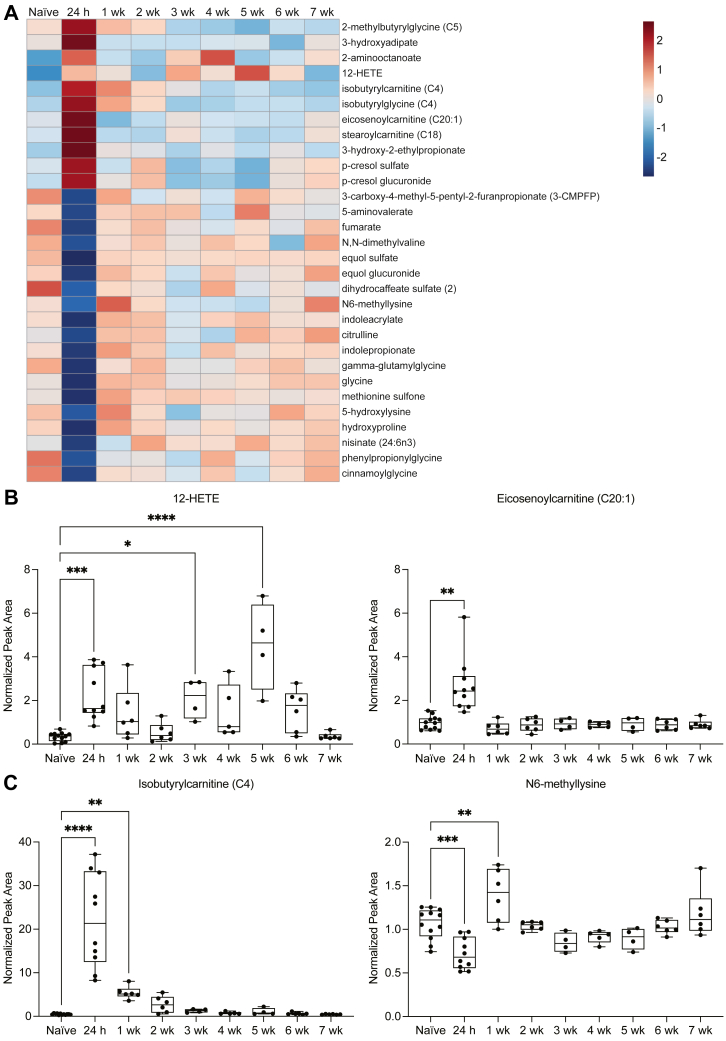
Temporal profile of the plasma metabolome after stroke in aged mice. A: Heatmap depicting group averages of the top 30 plasma metabolites altered 24 h after stroke compared to naïve controls. B and C: Lipid metabolism, represented by 12-HETE and eicosenoylcarnitine (C20:1), and amino acid metabolism, represented by isobutyrylcarnitine (C4) and N6-methyllysine, are dysregulated for at least 24 h after stroke in aged mice (12-HETE, N6-methyllysine: n = 4–12; ANOVA, ∗∗∗∗*P* < 0.0001; Dunnett’s multiple comparisons tests, ∗*P* < 0.05, ∗∗*P* < 0.01, ∗∗∗*P* < 0.001, ∗∗∗∗*P* < 0.0001; eicosenoylcarnitine (C20:1): n = 4–12; Kruskal-Wallis test, ∗∗*P* < 0.01; Dunn’s multiple comparisons test, ∗∗*P* < 0.01; isobutyrylcarnitine (C4): n = 4–12; Kruskal-Wallis test, ∗∗∗∗*P* < 0.0001; Dunn’s multiple comparisons test, ∗∗*P* < 0.01, ∗∗∗∗*P* < 0.0001). Data are presented as box and whisker plots, with boxes extending from the 25^th^ to 75^th^ percentiles and whiskers extending from the minimum to maximum values.

To assess whether the acute signature of dysregulated lipid metabolism was sustained in the weeks after stroke, additional plasma samples were collected at 1 week, 2 weeks, 3 weeks, 4 weeks, 5 weeks, 6 weeks, and 7 weeks after stroke in aged (20- to 23-month-old) male mice. These samples were also sent to Metabolon, Inc. for global untargeted metabolomic analysis using the Global Discovery Panel. To visualize the temporal profiles of the top 30 metabolites altered 24 h after stroke, the hierarchical clustering heatmap in Fig. 4B was expanded to include the subacute and chronic phases after stroke. The resultant heatmap demonstrates that the acute alterations in the plasma metabolome 24 h after stroke are transient; within 1 week after stroke, most plasma metabolites have reverted to baseline levels, with some exceptions (Fig. 5A). Interestingly, 12-HETE, an eicosanoid derived from arachidonic acid, exhibited a biphasic temporal profile, with acute elevation at 24 h and chronic elevation at 3 weeks and 5 weeks after stroke; contrastingly, eicosenoylcarnitine (C20:1), a monounsaturated acyl carnitine involved in fatty acid metabolism, exhibited a monophasic temporal profile, with transient elevation occurring 24 h after stroke (Fig. 5B).

To evaluate amino acid metabolism in the plasma after stroke, individual analyses of isobutyrylcarnitine (C4) and N6-methyllysine were performed. Isobutyrylcarnitine (C4), an acyl carnitine intermediate generated following the breakdown of valine, a branched-chain amino acid (BCAA), was abundantly elevated in the plasma 24 h after stroke and remained elevated until 2 weeks. Surprisingly, N6-methyllysine, a methylated form of lysine, an amino acid, was reduced in the plasma 24 h after stroke but was elevated at 1 week. By 2 weeks after stroke, N6-methyllysine had returned to baseline levels (Fig. 5C). These results indicate that amino acid metabolism is altered in the acute and subacute phases after stroke, exhibiting a monophasic paradigm that differs considerably from the biphasic paradigm exhibited by metabolites involved in lipid metabolism.

### Temporal profile of the plasma metabolome in the subacute and chronic phases after stroke in aged mice

**Fig. 6:**
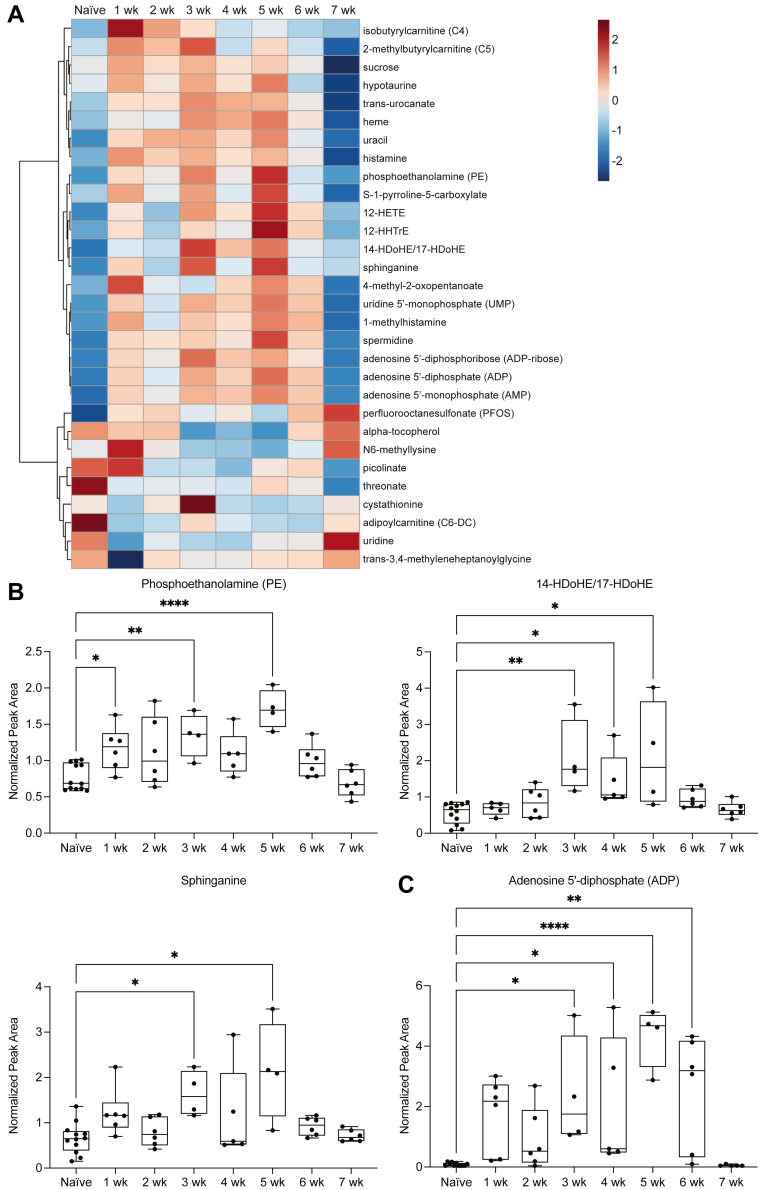
Temporal profile of the plasma metabolome in the subacute and chronic phases after stroke in aged mice. A: Hierarchical clustering heatmap depicting the top 30 plasma metabolites ranked by ANOVA (Euclidean; Ward). Isobutyrylcarnitine (C4), 12-HETE, and N6-methyllysine represent novel plasma biomarkers in the acute, subacute, and chronic phases after stroke. B and C: Lipid metabolism, represented by phosphoethanolamine, 14-HDoHE/17-HDoHE, and sphinganine, and nucleotide metabolism, represented by ADP, are significantly elevated in the subacute and chronic phases after stroke (phosphoethanolamine, ADP: n = 4–12; ANOVA, ∗∗∗∗*P* < 0.0001; Dunnett’s multiple comparisons tests, ∗*P* < 0.05, ∗∗*P* < 0.01, ∗∗∗∗*P* < 0.0001; 14-HDoHE/17-HDoHE: n = 4–12; Kruskal-Wallis test, ∗∗∗*P* < 0.001; Dunn’s multiple comparisons test, ∗*P* < 0.05, ∗∗*P* < 0.01; sphinganine: n = 4–12; Kruskal-Wallis test, ∗∗*P* < 0.01; Dunn’s multiple comparisons test, ∗*P* < 0.05). Data are presented as box and whisker plots, with boxes extending from the 25^th^ to 75^th^ percentiles and whiskers extending from the minimum to maximum values.

To further assess the subacute and chronic plasma metabolomes in an unbiased manner, a hierarchical clustering heatmap was constructed to depict the top 30 metabolites ranked by ANOVA and included plasma samples from aged (20- to 23-month-old) male mice at 1 week, 2 weeks, 3 weeks, 4 weeks, 5 weeks, 6 weeks, and 7 weeks after stroke and from naïve mice. With the exclusion of plasma samples collected 24 h after stroke, we were able to determine which metabolites were elevated or reduced specifically in the subacute and chronic phases without bias of transient acute alterations. The resultant heatmap illustrates dynamic alterations in metabolites involved in lipid metabolism, including phosphoethanolamine, 12-HETE, 12-HHTrE, 14-hydroxydocosahexaenoic acid/17-hydroxydocosahexaenoic acid (14-HDoHE/17-HDoHE), and sphinganine, and nucleotide metabolism, including ADP and AMP. Notably, isobutyrylcarnitine (C4), 12-HETE, and N6-methyllysine were conserved in hierarchical clustering heatmaps of metabolites altered in the acute, subacute, and chronic phases after stroke (Figs. 4B and 6A). These metabolites represent novel plasma biomarkers for stroke recovery in aged mice. In analyses of individual metabolites, we determined that phosphoethanolamine, a phospholipid derivative involved in cellular membrane structure, exhibited a biphasic temporal profile akin to that of 12-HETE, with subacute elevation at 1 week and chronic elevation at 3 weeks and 5 weeks after stroke. 14-HDoHE/17-HDoHE, a docosanoid derived from the autooxidation of DHA, and sphinganine, a fundamental component of sphingolipids, were also elevated in the plasma at 3 weeks and 5 weeks after stroke (Fig. 6B). These results provide evidence that lipid metabolism is perturbed in both the acute and chronic phases after stroke, exhibiting a biphasic paradigm.

To determine whether nucleotide metabolism followed a synonymous pattern, an individual analysis of ADP was performed. We determined that ADP exhibited prolonged elevation in the plasma at 3 weeks, 4 weeks, 5 weeks, and 6 weeks after stroke (Fig. 6C).

## Discussion

NfL is a structural scaffolding protein expressed abundantly and exclusively in neurons. Upon neuroaxonal injury, NfL is released into the cerebrospinal fluid (CSF) and peripheral blood, underscoring its value in neurological disorders such as ischemic stroke, Alzheimer’s disease (AD), and multiple sclerosis (19, 20). Using a Simoa® NfL assay, we confirmed that NfL was significantly elevated in the plasma of aged male mice at 24 h, 1 week, 2 weeks, 3 weeks, 4 weeks, 6 weeks, and 7 weeks after stroke compared to naïve controls. Correspondingly, we also observed a positive correlation between plasma NfL levels and infarct volumes 24 h after stroke. Khalil *et al.* hypothesize that the prolonged release of NfL after acute neuronal injury could be attributed to persistent blood-brain barrier (BBB) breakdown or chronic immunological or inflammatory processes (20). To further assess chronic sequelae, Spearman correlation analyses were performed on infarct volumes 24 h after stroke compared to lateral ventricle and hippocampus volumes 7 weeks after stroke. We observed a positive correlation between infarct volumes 24 h after stroke and lateral ventricle volumes 7 weeks after stroke, which indicates that NfL not only reflects acute neuroaxonal damage but also correlates with long-term structural alterations within the brain. Importantly, lateral ventricle enlargement is associated with cognitive impairment in nondemented elderly persons (21).

Early after stroke onset in patients, there are marked alterations in circulating lipids (22, 23, 24, 25); however, these clinical studies are limited by conventional lipid profiling (i.e., targeted analyses of HDL-C, LDL-C, total cholesterol, and triglycerides, also known as triacylglycerols). We hypothesized that the plasma lipidome 24 h after stroke also consists of an abundance of lipids derived from the breakdown of myelin and other cell membranes. These lipids, originating from plasma membranes of glial cells and neurons or myelin sheaths surrounding axons, enter the circulation via the damaged BBB. Accordingly, we have demonstrated that chronic stroke infarcts amass CE, SM, and sulfatides in young adult and aged mouse models (12). Using targeted lipidomic analysis, we discovered an acute signature of brain lipid catabolism in the plasma of aged male mice 24 h after stroke, reflected by the elevation of SM and HCER lipid species. These complex sphingolipids are primarily present in two locations within the brain: (i) lipid rafts in neurons, astrocytes, and microglia and (ii) myelin sheaths surrounding axons (26). Therefore, the accumulation of these sphingolipids in the plasma 24 h after stroke signifies neuronal cell death (27). Importantly, SM and HCER have been implicated in neurodegenerative diseases, including AD, Parkinson’s disease (PD), and Lewy body dementia (LBD); HCER lipid species are abnormally elevated in the plasma of PD patients with cognitive impairment (28, 29, 30).

We also observed a substantial reduction in plasma TAG lipid species 24 h after stroke. Interestingly, Jain *et al.* addressed the triglyceride paradox in stroke survivors and concluded that low triglyceride levels in serum collected within 24 h of admission were associated with increased stroke severity, reduced functional outcome at discharge, and increased 3-month mortality. It is presumed that low triglyceride levels reflect poor nutritional status, which leads to adverse outcomes after stroke (31, 32). Therefore, it is plausible that the reduction in plasma TAG lipid species 24 h after stroke reflects a transitory period of inadequate food and water intake previously identified in mice subjected to intraluminal filament MCA occlusion (33).

Using targeted lipidomic analysis on plasma collected temporally after stroke, we determined that the acute alterations in the plasma lipidome were transient; SM, HCER, and TAG lipid species returned to baseline levels within 1 week. These results indicate that there is a distinct signature of brain lipid catabolism in the acute phase after stroke. Although neuroaxonal injury persists in the weeks after stroke, we hypothesize that partial restoration of the BBB and phagocytic uptake of lipid debris by resident microglia and infiltrating macrophages lead to a reduction in brain lipid species detectable in the plasma. More sensitive detection methods may reveal prolonged elevation akin to the sustained elevation of plasma NfL observed with the Simoa® platform.

To date, ischemic stroke pathogenesis has been associated with disturbances in various biological processes, including energy failure, excitatory amino acid toxicity, oxidative stress, apoptosis, and inflammation (34, 35). These processes involve a multitude of metabolites, the qualitative and quantitative expression of which constitutes the central focus of metabolomic analyses. Using global untargeted metabolomic analysis, we discovered acute alterations in lipid metabolism and amino acid metabolism 24 h after stroke. Specifically, we detected increases in acyl carnitines, such as eicosenoylcarnitine (C20:1) and stearoylcarnitine (C18). Acyl carnitines facilitate the transportation of fatty acids for mitochondrial fatty acid β-oxidation. We have previously shown that increased fatty acid metabolism occurs in the brain in response to degenerating myelin (36). Therefore, the accumulation of acyl carnitines in the plasma 24 h after stroke may signify the metabolism of myelin debris. These results are in accordance with several studies demonstrating elevated levels of acyl carnitines in the blood of ischemic stroke patients (37).

Using global untargeted metabolomic analysis on plasma collected temporally after stroke, we determined that alterations in amino acid metabolism were apparent 24 h and 1 week after stroke but returned to baseline levels by 2 weeks. We also detected alterations in lipid metabolism in the acute and chronic phases after stroke. These results are in accordance with our previous studies that revealed a biphasic cytokine response to ischemia in the mouse brain, with the second phase initiated between 4 weeks and 8 weeks after stroke. In these studies, we also discovered that the second phase of inflammation coincided with an accumulation of foamy macrophages, T lymphocytes, and intracellular and extracellular cholesterol crystals in the stroke infarct (11).

To reduce the bias of acute disturbances in the plasma metabolome, we performed a subsequent analysis on plasma collected temporally after stroke but excluded samples collected at 24 h. We discovered chronic alterations in lipid metabolism and nucleotide metabolism that emerged 3 weeks after stroke. Specifically, sphinganine, a precursor in the biosynthesis of complex sphingolipids, was elevated in the plasma 3 and 5 weeks after stroke; sphinganine has been previously identified as a diagnostic biomarker for ischemic stroke (38). We also observed a prolonged alteration in nucleotide metabolism; specifically, ADP was elevated in the plasma 3 weeks, 4 weeks, 5 weeks, and 6 weeks after stroke. The sustained presence of ADP in the plasma after stroke may reflect various underlying processes associated with the response to ischemic injury. ADP, generated via hydrolysis of extracellular ATP released following cell damage, induces interleukin-1β release from microglia and macrophages and mediates inflammation in the CNS after injury, infection, and ischemia (39). Additionally, ADP, released from activated platelets at sites of vascular injury, contributes to the repair of damaged blood vessels and tissues (40, 41).

**Fig. 7:**
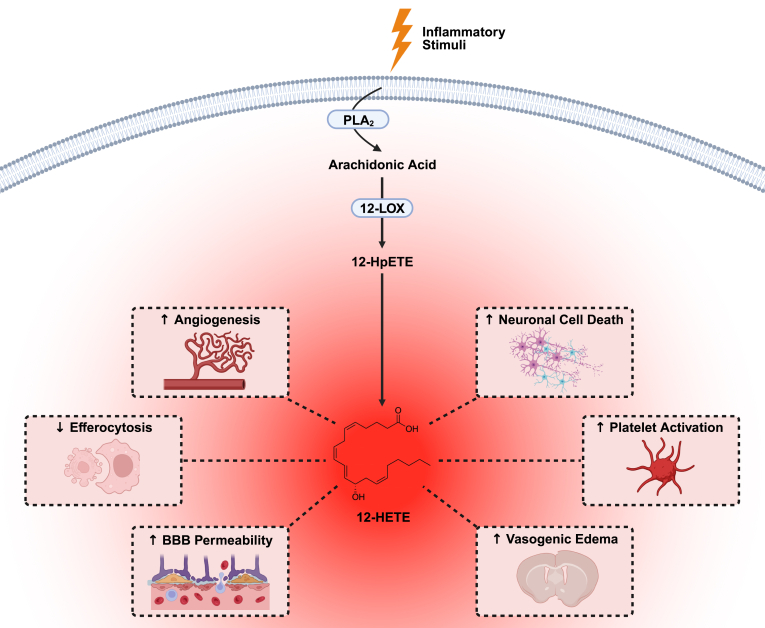
Biosynthesis and biological actions of 12-HETE. Arachidonic acid, released from cellular membranes, serves as the precursor for 12-HETE synthesis. The enzymatic conversion of arachidonic acid by 12-lipoxygenase (12-LOX), occurring predominantly in leukocytes, platelets, and vascular endothelial cells, results in the formation of 12-HETE. 12-HETE, a bioactive lipid mediator, acts as a signaling molecule in various physiological and pathological processes, such as angiogenesis, efferocytosis, and platelet activation, and plays a pivotal role in the inflammatory cascade by promoting leukocyte chemotaxis and adhesion to endothelial cells. Additionally, 12-HETE contributes to the synthesis of pro-inflammatory cytokines, exacerbating the inflammatory response. [Created with BioRender.com].

In these global untargeted metabolomic analyses on plasma collected temporally after stroke, we identified a novel plasma biomarker significantly altered throughout stroke recovery: 12-HETE. Arachidonic acid, released from cellular membranes upon encounter of inflammatory stimuli, serves as the precursor for 12-HETE synthesis. The enzymatic conversion of arachidonic acid by 12-lipoxygenase (12-LOX), occurring predominantly in leukocytes, platelets, and vascular endothelial cells, results in the formation of 12-HETE. 12-HETE, a bioactive lipid mediator, acts as a signaling molecule in various physiological and pathological processes, such as angiogenesis, efferocytosis, and platelet activation, and plays a pivotal role in the inflammatory cascade by promoting leukocyte chemotaxis and adhesion to endothelial cells. Additionally, 12-HETE contributes to the synthesis of pro-inflammatory cytokines, exacerbating the inflammatory response (Fig. 7). Correspondingly, Yoon *et al.* reported that 12-HETE was elevated in the plasma of young adult (10- to 12-week-old) male mice at 1 month and 6 months after transient MCA occlusion (42). We propose that 12-HETE, in addition to other bioactive eicosanoids derived from the arachidonic acid cascade (i.e., prostaglandins (PG), such as PGI_2_, PGE_2_, PGD_2_, and PGF_2α_, thromboxane A_2_ (TXA_2_), hydroxyeicosatetraenoic acids (HETE), such as 5-HETE, 8-HETE, 15-HETE, and 20-HETE, leukotrienes (LT), and epoxyeicosatrienoic acids (EET), such as 5,6-EET, 8,9-EET, 11,12-EET, and 14,15-EET), could function as plasma biomarkers of inflammation after stroke. Importantly, the biphasic accumulation of 12-HETE in the acute and chronic phases after stroke coincides with our previous studies, which demonstrated a biphasic pro-inflammatory cytokine response to ischemia in the mouse brain, with the second phase occurring between 4 weeks and 8 weeks after stroke (11).

In conclusion, we have validated that NfL is elevated in the plasma of aged (20- to 23-month-old) male mice for at least 7 weeks after stroke. We have also discovered an acute signature of brain lipid catabolism in the plasma 24 h after stroke. These lipids, including SM and HCER lipid species, could function as putative plasma biomarkers of neurodegeneration. In addition, we identified 12-HETE, a bioactive lipid mediator produced through the enzymatic oxidation of arachidonic acid by 12-LOX, as a putative plasma biomarker of inflammation. To our knowledge, extensive longitudinal lipid and metabolite profiling has not been previously reported or investigated in the context of stroke. These results offer valuable insights into the metabolic alterations that occur in the plasma after stroke and underscore the utility of myelin degradation products as biomarkers of neurodegeneration and arachidonic acid derivatives as biomarkers of acute and chronic inflammation. However, there are several methodological limitations that must be acknowledged. Firstly, these analyses were performed exclusively in aged male mice. Secondly, the systemic effects of temporal craniotomy and hypoxia on the plasma lipidome and metabolome were not explicitly addressed with sham-operated controls. Thirdly, due to sizable sample volume requirements for targeted lipidomic and global untargeted metabolomic analyses, in addition to plasma NfL quantification, mice were allowed to recover for a minimum of 2 weeks between blood sample collections; therefore, repeated measures were not employed. Future studies involving sham-operated controls and aged female mice are necessary to further characterize the systemic effects of temporal craniotomy, hypoxia, and sex on the plasma lipidome and metabolome. Additionally, there are inherent limitations to using plasma for biomarker discovery: (1) the difficulty in discerning the tissue(s) or organ(s) responsible for the observed alterations, (2) the intrinsic complexity and variability of biological fluids, and (3) the lack of sensitivity for detecting low-abundance proteins, metabolites, and lipids. To elucidate the origin(s) of metabolic alterations observed in the plasma, subsequent studies should include targeted lipidomic and global untargeted metabolomic analyses of brain and peripheral tissues, such as heart, liver, muscle, and lung.

## Data availability

The data described in this article are presented in the figures or supplemental materials. Additional data are available from the corresponding author upon request.

## Supplemental data

This article contains supplemental data.

## Conflict of interest

The authors declare that they have no conflicts of interest with the contents of this article.
